# Optimising recruitment to a late-phase tuberculosis clinical trial: a qualitative study exploring patient and practitioner experiences in Uzbekistan

**DOI:** 10.1186/s13063-021-05850-0

**Published:** 2021-12-04

**Authors:** Alexandra Wharton-Smith, Shona Horter, Emma Douch, Nell Gray, Nicola James, Bern-Thomas Nyang’wa, Jatinder Singh, Parpieva Nargiza Nusratovna, Zinaida Tigay, Emil Kazounis, Gulayim Allanazarova, Beverley Stringer

**Affiliations:** 1 Manson Unit, Médecins Sans Frontières, London, UK; 2 Clinical Research Department, London School of Hygiene and Tropical Medicine, London, UK; 3 Public Health Department, Médecins Sans Frontières-OCA, Tashkent, Uzbekistan; 4 Republican Specialised Scientific-Practical Medical Centre of Phtisiology and Pulmonology, Tashkent, Uzbekistan; 5 Republican Phtisiology Hospital No.2, Nukus, Uzbekistan

**Keywords:** MDR-TB, Clinical trial, Recruitment, Qualitative, Community engagement

## Abstract

**Background:**

Addressing the global burden of multidrug-resistant tuberculosis (MDR-TB) requires identification of shorter, less toxic treatment regimens. Médecins Sans Frontières (MSF) is currently conducting a phase II/III randomised controlled clinical trial, to find more effective, shorter and tolerable treatments for people with MDR-TB. Recruitment to the trial in Uzbekistan has been slower than expected; we aimed to study patient and health worker experiences of the trial, examining potential factors perceived to impede and facilitate trial recruitment, as well as general perceptions of clinical research in this context.

**Methods:**

We conducted a qualitative study using maximum variation, purposive sampling of participants. We carried out in-depth interviews (IDIs) and focus group discussions (FGDs) guided by semi-structured topic guides. In December 2019 and January 2020, 26 interviews were conducted with patients, Ministry of Health (MoH) and MSF staff and trial health workers, to explore challenges and barriers to patient recruitment as well as perceptions of the trial and research in general. Preliminary findings from the interviews informed three subsequent focus group discussions held with patients, nurses and counsellors. Focus groups adopted a person-centred design, brainstorming potential solutions to problems and barriers. Interviews and FGDs were audio recorded, translated and transcribed verbatim. Thematic analysis, drawing on constant comparison, was used to analyse the data.

**Results:**

Health system contexts may compete with new approaches especially when legislative health regulations or policy around treatment is ingrained in staff beliefs, perceptions and practice, which can undermine clinical trial recruitment. Trust plays a significant role in how patients engage with the trial. Decision-making processes are dynamic and associated with relationship to diagnosis, assimilation of information, previous knowledge or experience and influence of peers and close relations.

**Conclusions:**

This qualitative analysis highlights ways in which insights developed together with patients and healthcare workers might inform approaches towards improved recruitment into trials, with the overall objective of delivering evidence for better treatments.

## Background

Multidrug-resistant *Mycobacterium tuberculosis* (MDR-TB) strains pose a growing global health threat, with an estimated 500,000 cases reported in 2019 alone, of which 78% showed resistance to both isoniazid and rifampicin [1]. Mortality rates for MDR-TB patients are high, with 350,000 deaths estimated globally each year, and the post-Soviet Union countries such as Kazakhstan, Uzbekistan, Kyrgyzstan and Tajikistan bear some of the highest burdens of mortality worldwide [1]. In Uzbekistan, TB incidence in 2017 was 44.6 per 100,000, of which rifampicin resistance was observed in 6.1% of new diagnoses and 32.7% of previously treated drug-sensitive patients [2].

**Fig. 1:**
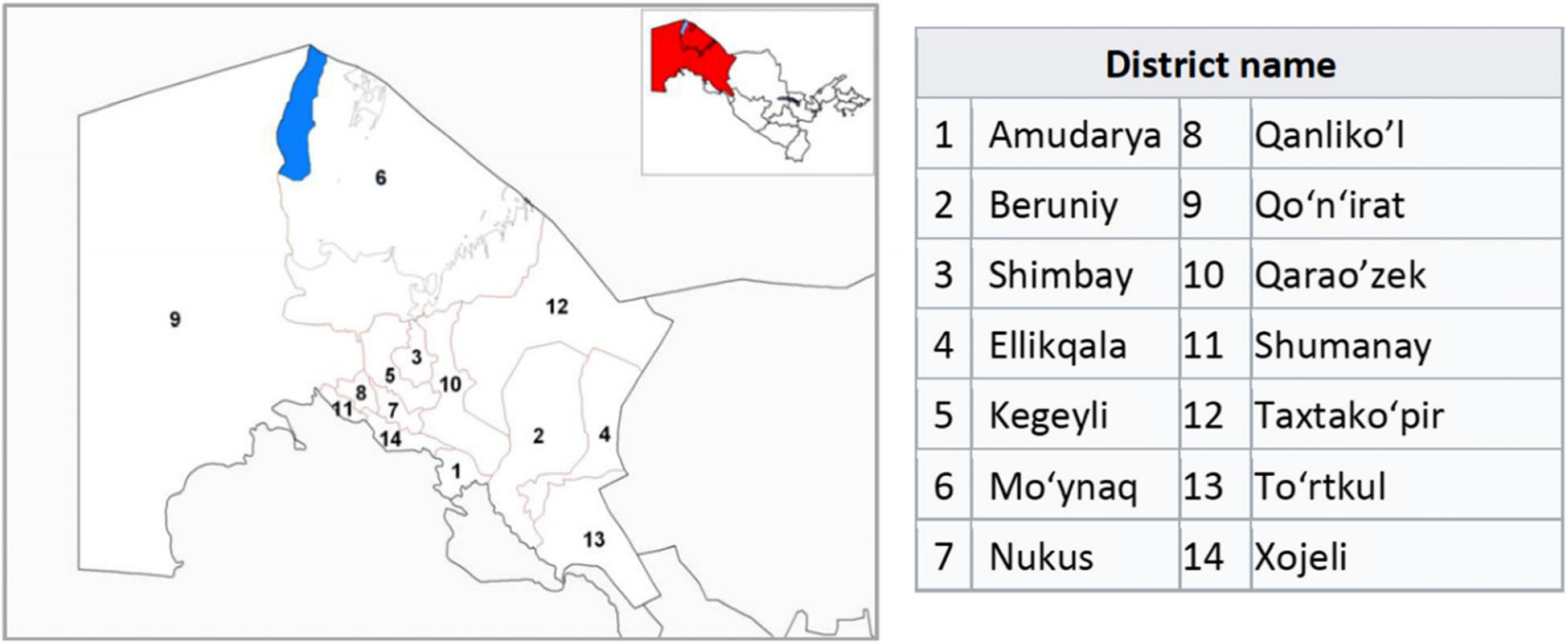
Map of Karakalpakstan and its 14 rayons

For the past 20 years, MSF has partnered with the Ministry of Health (MoH) in Uzbekistan to implement a comprehensive TB care-for-all programme, providing access to outpatient care, rapid diagnostic tests and a wide-ranging support programme for TB patients in the country. One specific area of the country, the Republic of Karakalpakstan, has some of the region’s highest rates of MDR-TB, with 41% MDR-TB in new and 78% in retreatment cases [3]. Karakalpakstan comprises fourteen districts or *rayons* (Fig. 1), with most of the region’s population of 1.7 million people living in the city of Nukus. Within Karakalpakstan, MSF has cooperated with Uzbekistan’s MoH and the Ministry of Health of Karakalpakstan, to deliver the TB-PRACTECAL study (NCT02589752); a phase II/III randomised control trial (RCT) for MDR-TB, examining the effects of new anti-TB medications, to find effective, shorter and more tolerable treatment regimens at six study sites across Belarus, South Africa and Uzbekistan. In Uzbekistan, the TB-PRACTECAL trial builds upon existing infrastructure in Nukus City, at the Republican Centre of Phthisiology and Pulmonology (TB1) and the Republican Phthisiology Hospital No.2 (TB2), where the first patient was recruited into the study in January 2017. The trial evaluates three novel treatment combinations for MDR-TB, all containing the new anti-TB medications, bedaquiline and pretomanid, assessed for non-inferiority against the standard-of-care (SoC) treatment locally. The three regimens compared in Phase II are (1) bedaquiline + pretomanid + linezolid + moxifloxacin, (2) bedaquiline + pretomanid + linezolid + clofazimine and (3) bedaquiline + pretomanid + linezolid. The most promising regimen will continue into phase III of the trial for further evaluation of safety and efficacy in a larger cohort.

The Nukus trial site in Uzbekistan has the largest potential catchment area for recruitment across the study. Yet, since 2017, accrual rates into the trial in Nukus have been around 20% of potentially eligible patients progressing to randomisation, which is both lower than expected and less than the other study sites; for example, in Belarus, the accrual rate was 35%. Preliminary anecdotal reflections from trial staff in Nukus suggested that local misconceptions and low research literacy in the community were potential factors for under-recruitment. These reflections led to the development of an operationalised, site-specific community engagement strategy; however, recruitment rates remain sub-optimal.

In general, recruitment to trials can be problematic, with only around 50% of clinical trials across clinical specialties closing having recruited to target [4, 5]; trial extensions are also commonplace [6]. Optimising enrolment to TB-PRACTECAL is vital to shorten the time to availability of any potentially successful treatment regimen. Increasingly, embedding social science methods within randomized trials is valued [7], particularly for enhancing consent procedures, understanding the impact of results, and highlighting contextual barriers to recruitment [8]. Patient preferences have also been shown to play a large role in decision-making processes during informed consent [9]. However, efforts to understand patient and practitioner perspectives in TB trials have thus far been limited. Data documenting these experiences is sparse, especially in Central Asia where the TB-PRACTECAL trial takes place.

The overall aim of this qualitative study is to understand patient and practitioner experiences and perceptions of recruitment to an MDR-TB clinical trial in order to optimise a patient centred approach to recruitment. The specific objectives of this study are as follows: (1) to identify factors which impede and facilitate recruitment, based on patient-participant and practitioner experiences; (2) to explore perceptions of clinical research among a sample of patient and health worker participants, and how this influences engagement with clinical trials; (3) to develop a patient-centred approach to recruitment (including process and tools) which optimises engagement with the trial, in collaboration with patient-participants and staff; and (4) to develop recommendations potentially applicable to other trial sites and countries conducting TB research in Central Asia and Eastern Europe.

## Methods

### Research design

We used a flexible participatory technique in which qualitative data were gathered with patients and practitioners using in-depth interviews and focus group discussions guided by semi-structured topic guides as well as field notes.

## In-depth interviews

Interview questions were based on themes relevant to the study aims and a literature search related to RCT recruitment. Following standard qualitative in-depth interview procedures [10–12], the order of questions was driven by the nature of participant answers, leading to the modification of the wording of the questions and the order in which they were asked during the interviews.

## Focus group discussions

We used FGDs to prompt selected cohorts of individuals to discuss shared experiences, perceptions and beliefs through mediated discourse. We tailored group composition and methodological design to the research question, with sessions guided by a skilled facilitator (AWS) and translators trained in qualitative methods to encourage open discussion and probe key themes. This was combined with person-centred design techniques, aiming to ‘meet people where they are’ to understand the behavioural factors that govern implementation of and compliance with interventions, products or services [13]. This uses a creative and systematic approach to problem solving, grounded in the context, emotions, needs and desires of key stakeholders for whom solutions are being developed [14]. These methods have been shown to enhance the use of theory-based and evidence-based approaches to intervention development, and to promote behaviour change [14, 15].

The co-design (interpretive) FGDs, conducted in January 2020 had two main goals, firstly, to brainstorm potential solutions to identified problems/barriers, including people, infrastructure, communication and material components of the recruitment process, and secondly, to co-develop specific solutions as prioritised by study participants and other key stakeholders, for example regarding communication materials, community engagement, training sessions, etc. [16]. FGD topic guides were informed by emergent themes in IDI data. In the FGDs, art media including sketching paper, pens, pencils, coloured markers and coloured plasticine clay were made available to participants who were invited to use these materials to create, visualise and communicate their ideas and suggestions.

### Setting and sample

The study was conducted under the joint MoH/MSF TB programme in 2019 in Nukus City, at the Republican Centre of Phthisiology and Pulmonology (TB1) and the Republican Phthisiology Hospital No.2 (TB2), Karakalpakstan, Uzbekistan.

IDIs included two key participant groups: clinical trial (CT) patients and healthcare workers (HCWs), respectively. Both patients and HCWs were sampled purposively, based on those who it was felt could provide a useful perspective on issues and questions arising from the analysis of routine recruitment data. We used principles of maximum variation, by selecting a wide range of dimensions of interest, aiming to discover central themes, core elements and/or shared across those selected. Snowball sampling was also used based on recommendations of participants to ensure inclusion of those who may be more sensitive to recruitment, for example linked to issues of stigmatisation. The sample size for each stratum was informed by theoretical saturation, the point of sampling at which no new, significant themes emerge in relation to the specific research questions [17]. It was estimated that a minimum of five interviews per participant group and one FGD consisting of a minimum of three participants should be achieved. Participants were not funded to participate in the interviews or FGDs. Light snacks and tea were served during each of the focus group discussions.

### IDIs with CT patients

Ten participants were selected from a full list of CT patients who had voluntarily consented to join the trial between 1 July 2019 and 31 December 2019, provided by the CT team. We aimed to include a range of ages, gender, TB treatment histories and access (inpatient, outpatient, outpatient home based care (HBC)), TB types (MDR/XDR-TB), geographic origins (rayon) and whether individuals accepted CT participation immediately versus experiencing multiple contacts with the recruitment team. One co-author (AWS) with experience in qualitative research reviewed the list, applying a purposive, maximum variation sampling approach, to select CT patients to represent a diverse sample. CT counsellors from the trial were consulted on the proposed list of participants to increase inclusivity of the sample, for example patients known to have experienced stigmatisation. Table 1 gives a descriptive breakdown of IDI participants sampled.

**Table 1:** Description of patient participants sampled

No.	Identifier	Patient type	Rayon (from)	TB type	Gender	Age
1	IDI01	Inpatient	Nukus	XDR	M	49
2	IDI02	Inpatient	Chimbai	MDR	M	51
3	IDI03	Inpatient	Khojeyli	MDR	M	19
4	IDI04	Inpatient	Nukus	MDR	F	27
5	IDI05	Inpatient	Nukus	MDR	F	34
6	IDI06	Outpatient	Nukus	MDR	F	45
7	IDI07	Outpatient (HBC)	Nukus	MDR	M	71
8	IDI08	Outpatient	Khojeli	MDR	M	37
9	IDI09	Outpatient	Nukus	MDR	F	37
10	IDI10	Outpatient (HBC)	Takhiatash	XDR	F	22

### IDIs with healthcare workers

We selected 16 healthcare workers, including MSF comprehensive care (CC) workers, CT doctors, nurses, and counsellors, MOH doctors and nurses and key CT staff at both urban and rural hospitals and clinics in Nukus and other rayons. We aimed to carry out IDIs with those workers who had been involved in recruiting high numbers of patients or were from clinics that had a high proportion of refusals. The interviewees sampled included 5 males and 11 females; 14 workers were national staff and 2 were expatriate staff; 2 sites were rural and 1 was urban.

### Focus group discussions

Based on a preliminary analysis of IDI data, three key homogeneous participant groups were recruited for the FGDs, promoting ease of discussion amongst peers, namely FGD1 including 10 CC and CT nurses; FGD2 including 6 CC and CT counsellors and FGD3 including 3 CT patients. These groups were identified as crucial to generating data on how CT recruitment could be improved. Participants are summarised in Table 2.

**Table 2:** Study sample size

Participant group	Data collection method	Number
CT patients	IDI	10
Healthcare workers	IDI	16
CC and CT nurses	FGD1	10
CC and CT counsellors	FGD2	6
CT patients	FGD3	3
**Total:**		**45**

### Data collection

IDIs were jointly conducted by the first author (AWS) and a CT translator in Karakalpak, Russian or English, depending on the participant’s preference, in a private a location for confidentiality, at an agreed convenient time and day. Interviews were audio recorded, then transcribed and translated verbatim into English, including local idioms. A random sample (five) of the translated transcripts was checked by a second translator for completeness and accuracy of meaning and translation. The audio recordings were then deleted to protect anonymity. One patient and four HCWs refused to be audio recorded but consented to the qualitative researcher taking notes during the interview.

FGDs were jointly conducted by the principal investigator (AWS) and a CT counsellor in the Karakalpak language as per the unanimous preference of the participants. All the FGD participants consented to the discussions being audio recorded. As with the interview data, the recordings were transcribed and translated into English.

All participants who consented to an interview were asked whether they would be interested in taking part in a separate, voluntary focus group discussion. All study participants were aged 18 years and above and gave informed, written consent to participate.

### Data analysis

Data from both sets of IDIs were analysed using an inductive approach, based on the principles of grounded theory, allowing research findings to be constantly compared and to emerge as themes inherent in the raw data [17]. The data was coded by the qualitative researcher (AWS) who had conducted data collection, and subsequently discussed with a trained anthropologist (BS) supervising the work, to identify emergent themes throughout the analytical process. A second qualitative researcher (SH) reviewed a sub-sample of transcripts and the coded data set, discussed the analysis with the first researcher and a consensus on themes was agreed and written up as the study results.

Data from the FGDs was analysed using an inductive approach, following the same process of identification of emergent themes and consensus among three members of the study team (AWS, BS, SH).

## Results

We present the main findings in two parts:

Part 1 introduces two main themes related to the importance of health system context in the implementation of a trial and the significance of trust in influencing engagement with the trial.

Part 2 describes the dynamics of patient engagement in the process of recruitment.

### Part 1: Health systems context

#### Systems and approaches in context of a clinical trial

This theme suggests that the health system context within which the CT is operating has implications for how new approaches may be perceived, particularly those aiming to investigate, test and implement new treatments for TB, and those with unknown outcomes. Certain participants described individuals, including MoH employees, becoming more familiar with the CT over time, which helped to build confidence in adopting a new approach, and this was reflected in their describing a sense of pride in contributing to better treatment and care for the future:

“In the beginning, for us also it was difficult to understand, because it is a new thing for MoH Karakalpakstan, MoH Uzbekistan even, and for us it was difficult to realize how the trial is and how we should start. We trained a lot and then compared with the first time, now for us it is easier to explain… I am proud to be a member of the clinical trial, it is a new thing… not only me, in general in the world, and it is ambitious project. I hope it will have good outcomes and it will be used for other people. I am proud that I give my contribution to this… outcome, and from our job we are giving people easier… less toxic treatment, for fewer months.” IDI26 (HCW)

The extent to which previous regulations and policies have played a role in the acceptance of new treatment and approaches may be relevant in this instance. Some HCWs described experiencing difficulty achieving patient compliance without such controls, whilst at the same time realising the disadvantages of compulsory treatment:

“[There is] compulsory treatment. But it seems it is not very efficient, even we have challenges, for example, even in the compulsory ward, there they don’t intake the drugs. Because you cannot force anyone… Compulsory treatment it’s a…it’s a law. Community TB doctor writes to the court name of the patient, that is smear positive and… transmitting the TB… which is fair I think… and then they, police coming and then taking, bringing the patient to the OPD. This compulsory ward.” IDI17 (HCW)

Tension may emerge within systems where two distinct practices coexist, for example, according to the CT guidelines, patients could be hospitalised for 14 days however, the local regulations or “*prikaz*” could supersede this and force the patient to stay longer, until they were smear negative. This created the perception of contradictory information which undermined trust in the CT.

“Another big challenge is that patients have been told that you have to be admitted to [hospital] for at least 14 days or something like that... if you tell someone ‘oh you are going to be admitted for 14 days,’ after 14 days they want to be discharged. But in [hospital] what happens is [they] really follow the prikaz… If the patient is smear positive, they can’t discharge the patient. Yea, so this becomes an issue. Because in the mind of a patient it’s 14 days, so not releasing after 14 days and then they blame the counsellor because ‘you lied to me’. Because patients won’t do that to the doctor. It’s very rarely that a patient says directly to the doctor ‘you are lying to me’, you know... Because of this, they don’t want to be admitted.” IDI24 (HCW)

“In TB 2 they don’t allow to go home till the end until they are discharged totally, in other places they can get permission to go home on weekends for one or two days.” FGD01, R8 (HCW)

Perceptions of workload and responsibility for patients were another issue highlighted between MoH systems and the CT. In relation to this, whilst MoH HCW may have appreciated being able to distribute their workload by recruiting patients into the CT, there could potentially be negative repercussions if they then had empty hospital beds within their own hospital wards, which could disincentivise MoH from collaborating with the CT.

“[There are] two regulations. For example, we might have our standard protocol and clinical trial protocol and MoH local protocol… Sometimes they don’t fit each other, and it contradicts. This is why, they prefer, if they are smear positive patients, they prefer to admit, admit to the IPD… Because they say that they will be fined.” IDI17 (HCW)

Competing dynamics within the health system may result in perceptions that lead to lack of referral of potentially eligible patients to the CT or poor attendance to joint consultation sessions during the CT recruitment process:

“Some other doctors see the clinical trial like somebody that is stealing their patients… they see that clinical trial, like they are little bit privileged, so they are jealous about it. And, yeah, this is also sometimes is also this part, in the past was also from the other side, the clinical trial doctor believe that they were better than the others… it was both sides. That was pushing on this thing. So they would feeling better, to treat the others with superiority. Here is the ex-Soviet Union country, so hierarchy is big thing. And the others feel like, ‘you don’t have the right to feel better than me.” IDI13 (HCW)

#### Significance of trust

The concept of trust appeared to be key to understanding individuals’ engagement with the CT. Linked to competing health systems previously described an unfamiliarity with the concept of “research” or clinical trials was expressed (Table 3).

**Table 3:** Quotes; trust and hope, fear and scepticism

**Trust and hope quotes**	
“I was not scared at the beginning...[of] six-month treatment. I mentioned that my mother was treated [with CT]. I agreed to be a part of clinical trial believing I would be cured if I take drugs without missing any drug. I am interested in it myself.” IDI04 (female patient)	
“Patients play crucial role in influencing each other… They trust each other even more than us [HCW].” FGD01, R7 (HCW)	
“Another thing that I think can help… they really trust and believe if they can speak with another patient that completed the trial. Of course, I think it is impossible, you create bias, I do not know. But if they can do that, I think that can help. Because they [patients] trust their peers. Because this is somebody who passed through and completed... So, if that was possible, I think the recruitment would go up very fast.” IDI13 (HCW)	
“They [patients] need to trust, they need to have confidence.” IDI06 (female patient)	
“I joined voluntarily, as I was sick. I thought I would be cured if I took this treatment. Even I did not discuss it with my husband. I believed I would take drug, and if I took drug, I would be cured. I am encouraging myself to continue with treatment.” IFI06 (female patient)	
“I agreed to be a part of clinical trial believing I would be cured if I take drugs without missing any drug. I am interested in it myself…It is written that in case there are side effects they [CT HCW] will treat themselves. Therefore, I trusted, we are getting better care now compared to the previous standard care.” IDI04 (female patient)	
**Fear and scepticism quotes**	
“People are afraid of the clinical trial… as research has never been done in Karakalpakstan before. This is something new for Karakalpakstan.” FGD01, R1 (HCW)	
“They might think that why they are being given clinical trial, why not standard treatment? They might think they are being used…how to say this…eh…they think they are being experimented [on].” IDI21 (HCW)	
“At the beginning, any person who comes here to join the clinical trial, before signing the consent, they will have thoughts – they do not want to be ‘used’ as it has the name “trial”. It is like a rabbit in an experiment; especially if that person is a patient. You know for the patient; everything is very sensitive.” IDI08 (Male patient)	
“There are patients who say that, ‘oh I don’t care if you are experimenting but standard of care is a tried and tested regimen and I prefer to go with something that is, you know, going to work for sure. I don’t know if your treatment is going to work or not.’” IDI24 (HCW)	
“There are kind of rumours like: ‘If you pay money they put you in [the short course arm]…’ Randomisation is not easy to understand.” IDI13 (HCW)	
“They came to TB2 and they saw other patients, they heard from them, maybe stories… and then they refuse to continue with CT.” IDI18 (HCW)	

For both patients and the HCW, it appeared particularly important to have experiential evidence that reinforced trust in the CT. For HCWs, seeing patients achieve good outcomes could instil confidence in the CT, and such experiential evidence may be more important than scientific evidence or information. One HCW, who did not consent to quotes being used, described MoH doctors’ main concerns with the trial regimen to be around effectiveness—wanting to see successful outcomes for patients in order to be able to believe in the CT and support it. This could be a recruitment bottleneck for the trial, as data could not always be shared with HCWs, and some requested more transparency around preliminary findings.

The concept of trust emerged as an important issue within patient participants’ accounts of their willingness to engage with the CT. The importance of having hope, belief and confidence in the treatment and the potential to be cured were described. Trust appeared to be imbued within practitioner-patient relationships, with patients largely deferring to their doctor to decide on the appropriate treatment course. Doctors were seen as having greater knowledge and experience and therefore were considered best placed to make treatment decisions. Trust therefore appears to be key in relation to understanding individuals’ engagement with the CT. Within this setting, there is unfamiliarity with the concept of “research” or clinical trials, with old and familiar ways to approach treatment thought of as more reliable than unproven treatments.

#### Fears and scepticism

Fears were described relating to the novelty of the CT, which in this context was said to be an unknown concept, with low biomedical and research literacy and unfamiliarity with clinical trial research reportedly common (Table 3). Patients were said to have fears about why they were being treated differently from others, being offered the trial when most receive treatment within standard health services. Fears were noted in relation to the more rigorous health checks involved in the trial process. Doubts were also described relating to CT drugs being presented as the “last option”, which could deter some from engaging with the CT due to concerns of potential relapse in the future.

Interview participants referred to the trial as an “experiment”, with suggestions that it was being used to test foreign drugs, rather than to improve treatment for the population of Karakalpakstan. This was related to the limited terminology available to describe the CT in the local language of Karakalpak, where the terms “research”, “clinical trial” and “investigation” can only be communicated using one word “*izertlew*”. Translated into Russian, “clinical trial” can be translated to “experiment” (‘*espetania*’; “пробный”, “эксперимент”). Unfamiliarity and misunderstanding of the CT appeared to contribute to scepticism, confusion, rumours and trial refusal. Over time, becoming familiar with the treatment through seeing others improve appeared to calm and reassure such fears.

**Fig. 2:**
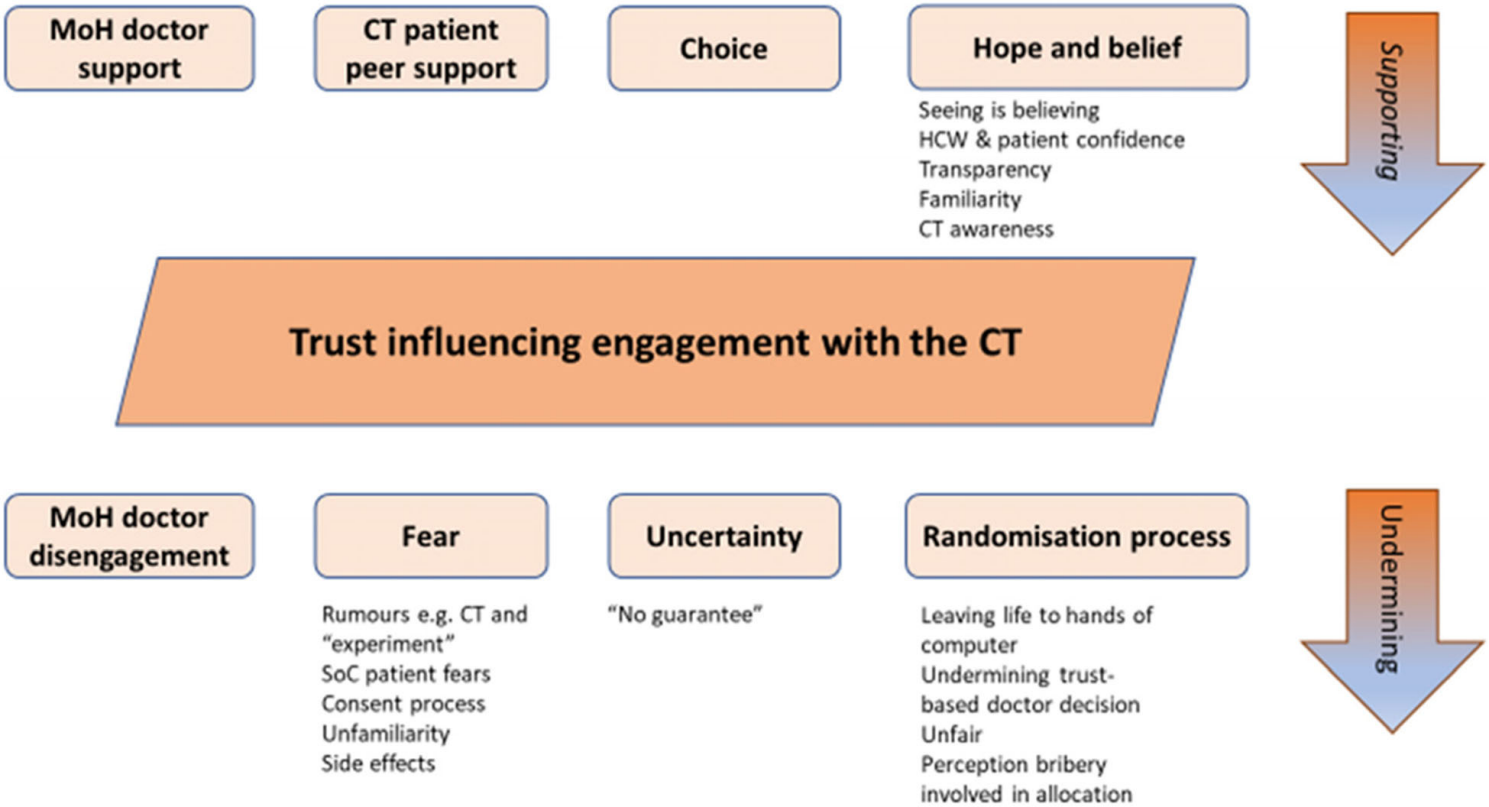
Schema illustrating how aspects relating to trust may influence CT recruitment

The schema shown in Fig. 2 presents how the theme of fear and scepticism may undermine trust in CT engagement, whilst hope and belief may foster it.

#### Randomisation

The randomisation process appeared to exacerbate some patients’ fears and uncertainty. Many were said to misunderstand randomisation and described beliefs that treatment allocation was based on bribery, which appeared to contribute towards trial distrust. Randomisation was stated to be unfair, and some interviewees reported it was difficult for patients to accept their allocated treatment assignment, particularly for those allocated to the longer treatment regimen. Some patients appeared to take a deterministic approach to understanding the randomisation process, interpreting the treatment allocation as ‘God’s will’. Additionally, the randomisation process appeared to counter the more familiar process of a doctor deciding on the appropriate treatment option, which again could counter trust in the CT. However, some patients did appear to understand the randomisation process, describing the decision as being “helped” by a computer, which may support their engagement with the trial.

#### Evidence that it works

As is inherent within clinical trials [18], there is a large amount of uncertainty regarding the effectiveness of the included regimens and the potential side effects, which can exacerbate fears and undermine CT engagement. The CT was described as having “no guarantee” and was perceived as less reliable than the standard regimen. Patients were also said to have fears about potentially wasting time and needing to start treatment again if the trial drugs do not work.

### Part 2: Processes around engagement in the trial

**Fig. 3:**
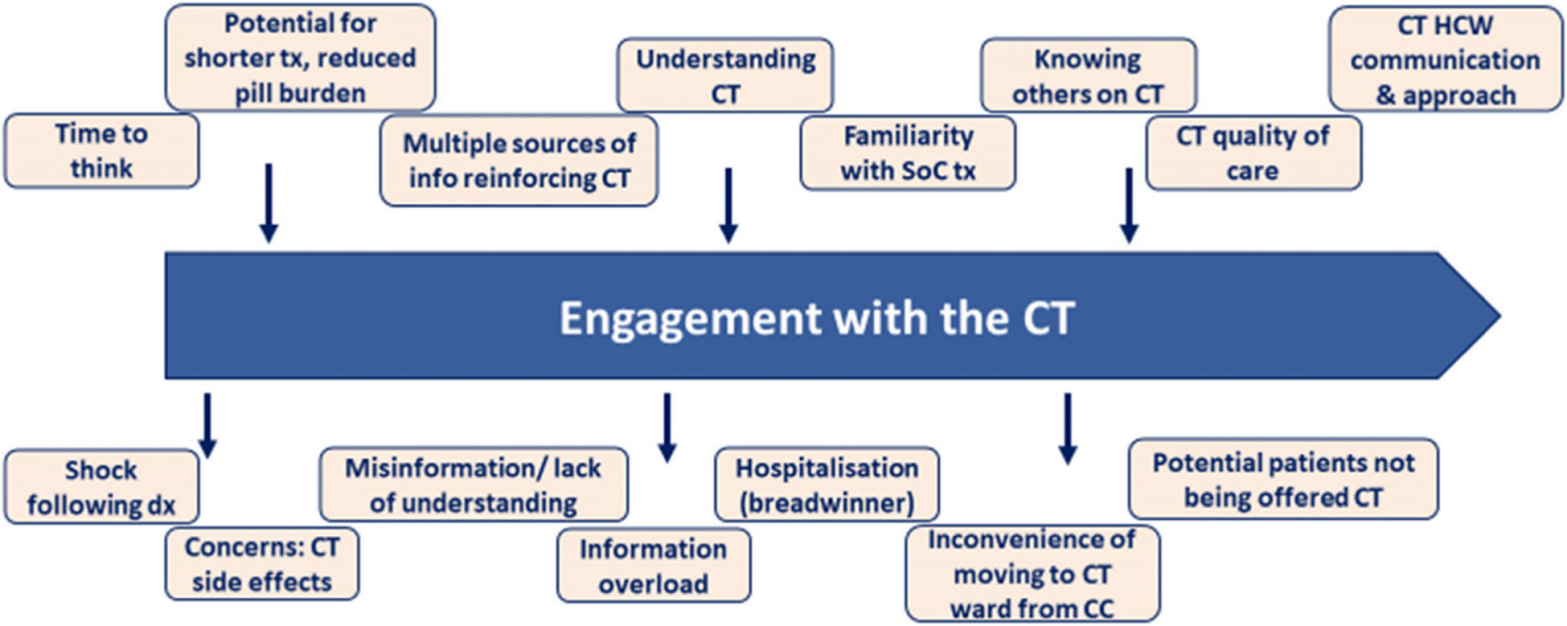
Schema illustrating the dynamic processes in trial recruitment

**Fig. 4:**
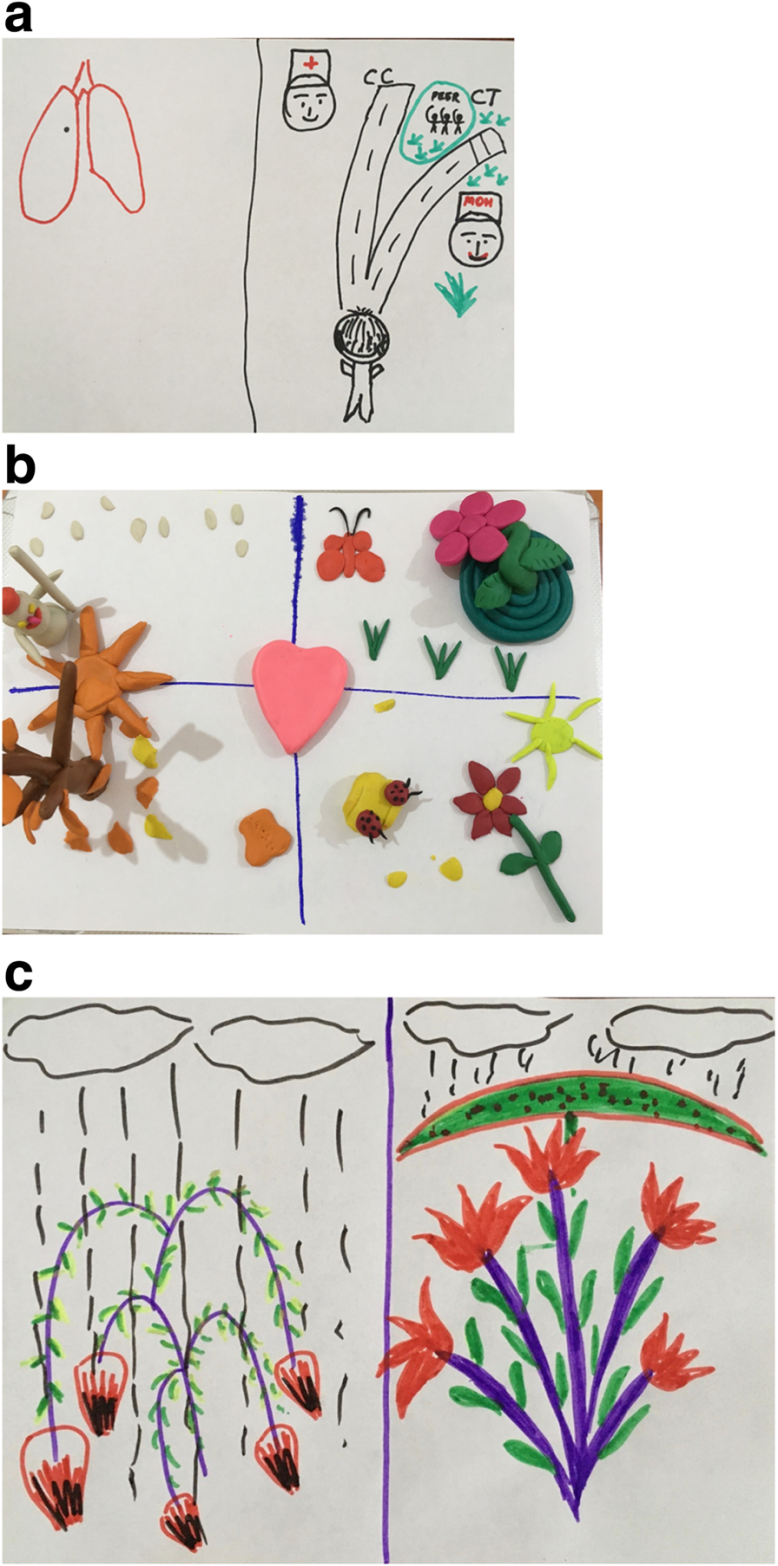
**a** Drawing that illustrates the decision paths “CC” (Comprehensive Care) or CT (clinical trial) for a TB patient, HCW, FGD. **b** Sculpture depicting how community engagement activities should be held in all seasons (FGD, HCW). **c** Drawing that illustrates how patients suffer from TB (on left) and blossom under CT care and treatment (on right) (FGD, HCW)

The decision-making process for individuals regarding whether to engage with the CT are non-linear, independently and temporally varied. Various factors can influence engagement with the CT, driving and undermining it (Figs. 3 and 4a).

#### The dynamics of the CT decision-making process

Many individuals reported experiencing shock on receiving a TB diagnosis, which was noted as lessening their ability to receive and process information at this time.

“At the beginning I had no idea about it [TB]. Then counsellor came, translator came and Dr X [foreign doctor] came, and also Doctor Y [MSF CT Doctor] came. I was still in shock at that time; actually, we do not understand what is TB. There is a saying like: ‘if you do not get sick, you will not know about it.’ I had a bad understanding. I was feeling strange [uncomfortable]. They were 3 or 4 sitting here and [they] explained about the clinical trial.” IDI08 (male patient)

“I went there, entered, there was a doctor. I greeted her, she invited me to have a seat, and then she told me that I have bad results of analyses. It was conveyed to me at once, unexpectedly and I got lost… I didn’t know what to do, I was just shocked. Doctor doesn’t know how to communicate with patients… When I was informed that I have bad results I remembered about my father’s case. Because my father died of this disease. The first thing I thought about was that I would die anyway [crying].” FGD03 R3 (female patient)

It was said to be important to have time to process the information about a TB diagnosis and to consider information relating to the CT. Several participants described appreciating the opportunity to take the time available (up to 14 days were described as allowed) to consider whether they would like to join the trial, though this varied, and some reported wanting to join sooner.

“I did not come to conclusion very fast; I thought about it a lot for 5-10 days.” IDI08 (male patient)

Many patient participants described consulting multiple sources of information after the initial trial explanation and offer, including discussing the CT with others, reading information, wanting to speak to other patients, wanting to see the trial space and seeking information. Where these sources of information reinforce the CT message, and anticipated benefits, this seemed to support engagement with the trial:

“I read the materials that they gave me. I read all of them very carefully. I also came here to check how this clinical trial going on. I talked to some patients before I gave my consent. They waited for me for some time around 10 days. I called the patients and I asked them about treatment and other things. They explained me about clinical trial with their language. Depending on their horizon. I tried to find out more information from everywhere then made a conclusion to join here. I came here to see how everything was and what would happen in the hospital. Where the hospital was or how the condition was there? Then I came here [to CT].” IDI08 (male patient)

“After patients have this session, many often go off on their own to find out more information about TB, and what the standard care consists of.” IDI11 (HCW)

#### Collective decisions and influence of peers

Certain participants described discussing their decision with others, for example seeking the opinion of their family members. However, others described coming to the decision on their own. There appeared to be gendered aspects to decision-making, and certain married women (“daughters-in-law”) were described as having less autonomy for such decisions, relying on the decision of their husband or mother-in-law. For such individuals, it may be important to involve the patients’ family members in the consultation and discussions about the trial.

“It is difficult to explain about the clinical trial to everyone. For example, the wife may want to join the clinical trial, but the husband can be against it [not allow her]. So, it’s important to include the whole family in the consultation. Usually men have more power over women here. In-laws can also be the ones who make the decision.” IDI20 (HCW)

“It’s way more complex in the case of women, because… the husband gets involved, the mother-in-law gets involved, the father-in-law gets involved and then the whole family gets involved… for a married woman especially. And it becomes way more complicated because then the family has a lot of questions of their own and it takes more time to start… for them to join.” IDI24 (HCW)

However, this will not be the case for all married patients, as one 45-year-old married patient said:

“I did not discuss my decision with my family… Even I didn’t discuss with my husband… I did not think about anything except being cured.” IDI06 (female patient)

#### Knowledge, familiarity and understanding of the CT

Understanding of the CT appeared to be a key factor influencing engagement with it. Within this context, individuals are described as having low awareness about TB generally, including relating to TB aetiology and transmission. The trial recruitment process was said to contain a large amount of complex information, with a heavy reliance on written information, which may be difficult for many to process and understand. A lack of understanding about the trial process, risks and benefits was viewed as undermining engagement:

“People need more information; they need to have awareness sessions, if they know more information, they will join CT. It is better than previous standard of care. The condition is good; care is good. However, I think… most people are interested in it, but… mmm… it is not developing. Some people do not understand, they think that it is hard; they are scared. Their awareness needs to be increased.” IDI04 (female patient)

“It also depends on the awareness of people about TB. If a patient knows something about TB, in that case they start getting interested in it [CT]… I think it might depend on the ability of people to think, [it] also depends on how knowledgeable people are.” IDI12 (HCW)

Certain HCW described patients’ education level as influencing their ability to process CT-related information, and their likelihood of having a positive or negative reaction to the trial (with those of lower education level perceived as more likely to have a negative reaction to the CT):

“They have different reactions. If the patient is educated then they will show interest and start asking questions. If the patient is not educated or have low level of education, or the patient who does not have any information about TB, when they hear about trial, of course they will have a negative reaction at the beginning.” IDI19 (HCW)

Understanding was said to be facilitated using visuals, and patients described appreciating the counsellors’ support and the opportunity to discuss the CT and ask questions. Such visual tools appeared to support the processing of information and understanding, whereas the reliance of written information could favour more literate patients and could also be quite overwhelming:

“It is better if the information is explained. They used computer and visual aids to explain, after that, I understood. Initially they left paper to read, but I couldn’t get much out of it. After they explained thoroughly, I was able to get the full picture. I think it will probably be preferable way to other people as well.” IDI05 (female patient)

“No, all information was enough. It was clear... Verbal one [information was more useful than written one]. They showed me video on a computer. That one was useful. Everything was shown here before being hospitalized. Everything was enough, mostly they explained, I read information as well. I was given a brochure. Everything was provided. We had verbal discussion as well.” IDI06 (female patient)

Increasing appreciation about the CT among community members was stated as important for supporting recruitment, as understanding and awareness could help to normalise perceptions of the trial and counter fears. Additionally, having awareness and understanding of the CT prior to diagnosis may facilitate individuals’ ability to decide on their participation, as shock at the time of diagnosis can undermine their processing of information. Participants felt that fears relating to drug side effects could be reassured through knowledge of the rigorous checks and support available in the CT and that it is important to inform people of the strategies included in the CT to mitigate risks. Certain HCWs felt that CT information could be updated to reflect potential risks of adverse reactions, including relating to the risk of heart attack, in line with current research:

“It would be better if there are more conversations… When the patient comes, anyway, they are scared of lung disease; they have a fear. At that time, it is preferred to have more conversations. There should be more talks in policlinics, if people get more information and become aware, they may understand it… people need more information, they would like to know more… For example, in this clinical trial, even though it is said that there are side effects, you have ultrasound investigation done, if your lung or other body parts have pain. There are some people… with low income [who] have difficulties, so they need more awareness sessions.” IDI04 (female patient)

“I think that the point about heart stop might be changed, because so far there has not been any case in which heart has stopped working. Also, and it has been used for 3 years, Pretomanid is used in other trials also, clinical trials are conducted in other places as well. If there are changes, they might be integrated.” IDI12 (HCW)

Participants suggested having more regular community engagement sessions, held at least quarterly, and highlighted the need for a specific health promotion resource for this, including the recruitment of staff members (Fig. 4b). It was felt important that engagement and sensitisation activities extend beyond the TB setting, to include community structures, broader clinics, and health system structures (including policlinics and community/family doctors), and schools. Informal discussions and questions were preferable to formal lecture-based sessions. Additionally, the use of drama and social media was recommended to relay information to the community. Participants felt that this sensitisation should incorporate TB generally, as well as the available treatment options, including the CT.

“It is not enough to learn about it in TB facilities, but in other places as well as local committee facilities they can hear, see about it, and then they can share this information.” FGD02, R1 (HCW)

Participants felt that increased awareness may also support CT recruitment in reaching and informing all eligible patients. Examples were given of where patients were not currently informed about the trial, and thus miss the potential to be recruited:

“I learned [about CT] after I was hospitalized in the diagnostic ward, before I had not known about it… In order to inform the population about it earlier, something should be done. As in my case, if I had known earlier, I would have got treatment earlier. In addition, about clinical trial, I learned recently. Before I was doing self-treatment.” FGD03 (patients)

#### Setup and quality of TB care

HCW reported feeling that some patients did not want to participate in the trial due to the obligatory 2-month hospitalisation period required within the CT. This has reportedly improved since this mandatory hospitalisation was reduced to 2 weeks in September 2019. However, this still poses a challenge for patients who live further away from trial sites, or who are responsible for securing an income and providing for their family, often the case for men. Additionally, the inconvenience of having to change wards, from CC to CT, once admitted was described as a potential barrier to recruitment:

“Some patients… they want to participate after the first session, but some of them refuse it because they are already admitted, so patients who are on treatment CC - Comprehensive Care, they want to…they do not want to change ward, doctors, treatment and they want to stay in the same ward.” IDI18 (HCW)

“I was in hospital for 2 weeks because the doctor told me that I had to be there. I do not have time. Because I am the only man at home. I am the breadwinner at home. I do the business myself that is why I have to be here.” IDI08 (male patient)

Perceived benefits of the trial were described, which appeared to support engagement with it. These included the potential for enrolling on a shorter treatment regimen, and hopefully with fewer side effects:

“What I like in clinical trial, for example in SoC the treatment is 24 months, you come and go for 24 months, it is very difficult. But, here if God wishes, you get treated in 6 months.” IDI08 (male patient)

“They [patients] want to decrease the treatment and pills, the number of pills… it will be very… I would say it will succeed… it will be a revolutionary way of treatment, cutting from 24 months to 6… it’s like 4 times less and less pills… it’s a very big thing actually” IDI17 (HCW)

One of the most commonly described potential benefits of the CT, which was reported to support individuals’ decisions to engage with it, was the perceived superior quality of care with the CT than the SoC (Fig. 4c). This was described in terms of the thorough medical examinations and care, the cleanliness of the ward, HCW’s communication and approach and supportive practitioner-patient relationships. Several patients spoke of valuing and appreciating HCW’s courteous and caring approach, which was perceived as being better among CT staff than their experiences with MoH HCW and which supported their choosing the CT:

“What I liked in clinical trial that everything was under control and clean. Everything is clean here. It is not just simple words. I like everything here, nurses’ attitude, and doctors’ attitude… I came here with my consent and what I like here is that everything is done in a timely manner. You come here and tell them everything. If you want to talk to the counsellors, they tell you the time and date. If it is at 10 o’clock then I come at that time. We sit and talk. Now I am taking my pills and my health is fine. I feel well.” IDI08 (male patient)

“It seems there are a lot of advantages here when you get treatment. The doctor and patient relationship is good. At the beginning, they did a lot of medical examinations per week. They examined my whole body. It is interesting to an old man who understands. They examined my heart, ears, and eyes and did specimen tests. I am interested in medicine.” IDI07 (male patient)

Additionally, one patient appreciated that the CT maintained anonymity:

“[MoH doctor said] you will take this treatment minimum 9 months as it is a standard treatment, or two years. She did not tell me about this trial. Then I asked support from her. Let us find a way, so that people will not know about my treatment. She asked what way. Then she had an idea and mentioned about clinical trial… Here in clinical trial, even they do not tell your name. They call me [patient number], come in please, or something else. It is good and I like it.” IDI08 (male patient)

## Discussion and conclusions

Our findings highlight the consequence of social and healthcare systems within the context of implementing randomized control trials. Where approaches are largely unfamiliar to patients, and familiar treatment were thought of as more reliable, this led to a reluctance to try “unproven” treatment regimens, which meant that patients needed time to develop confidence around new approaches. Confidence in the trial seemed to be supported by having direct experience over scientific data; this also applied to healthcare workers, whereby doctors reported wanting to see their own patients achieving good results. Two co-existing health systems and approaches (CC and CT) give rise to competing interests and a lack of acceptance among both staff and patients. Over time, an increased awareness from information sharing through trusted channels fostered a positive perception and acceptance of the trial.

Direct support from MoH doctors and understanding the randomisation procedure appeared to be important influencing individuals’ engagement [19, 20]. Increasing CT familiarisation, involving CT patients in communicating with potentially eligible patients if possible, and explaining the CT could address and reassure the perceived widespread fears. Misunderstandings could be supported by admitting potential CT patients to the CT wards rather than CC ward, as well as through increasing a welcoming and supportive process. As Scott et al [21] mention, any faith that draws a patient into a trial will be strengthened during their experience by such things as behaviour and responsiveness of the practitioner, each of which makes volunteers feel highly valued.

Patients’ hopes for shorter, more tolerable treatment and the perceived superior quality of care in the CT in comparison to the SoC were noted to positively influence their engagement with the CT. Trust in the CT could be supported through positive peer-to-peer information, e.g. seeing family members enrolled into the CT, seeing the CT ward or having contact with other CT patients who share their experiences and know the reality of the trial [21]. Similarly, patients’ ability to process information relating to the CT, and trust in the CT, were supported by exposure to empirical evidence and familiarity, e.g. being familiar with TB treatment and the existing options. Our data illustrate that as time went on and participants and HCWs became increasingly familiar with the trial, and were exposed to patients’ positive experiences, fears around the trial were to some degree assuaged [22].

Quality of communicating knowledge or familiarity with the CT prior to a patient’s initial TB diagnosis could support their ability to process information related to the trial. Additionally, individuals may seek and consult multiple sources of information in their decision-making, including seeking other HCW’s opinions, particularly a trusted doctor who may be their family/community/MoH doctor, reading and discussing the CT with other patients. Increasing the quality of the partnership with clinical trial team is known to contribute to patients remaining in a clinical trial [23]. Where relevant, inviting husbands and/or mother-in-laws to participate in receiving information and explanations about the CT might also support engagement, as key individuals reported to be involved in some women’s decision. Visually relayed information appeared preferable to written information, and the written consent form may contribute to fear and refusal.

Optimising recruitment requires enhancing trust in the trial through building familiarity and understanding, addressing fears and concerns, specifically the randomisation process, sharing preliminary results with HCW where possible, and supporting patients’ access to see the CT ward and contact with other CT patients. Uncertainty about randomisation can then decrease as familiarity with the trial increases. Building upon successes and promoting the reputational high quality of care and compassion is also key. This means that the recognition of systems as well as processual barriers need to be addressed through changing key activities and approaches, as highlighted in (Table 4). Practical implementation requires devising these with the team and participant groups.

**Table 4:** Key findings and implications for practice change

Activity or approach	Advice for change	Examples	Practice
CT awareness and familiarity	• Broader awareness of TB, treatment options and CT • Information to be consistently talked about beyond the point of diagnosis	• Ongoing active community engagement activities • Health promotion team participation in established community structures • Information to be consistently talked about within policlinics • Use of positive films • Use of social media	• Community engagement to include spectrum from informing to collaborating [24, 25] • 2-way peer approach to tackling issues raised on understanding and info circulation
Addressing fears and concerns	Information and support, e.g. to explain and reduce fears of CT as “experiment”, concerns about side effects	• Emphasise drug safety/approval, accountability, and mechanisms of responsibility, monitoring of side effects, etc. • Build awareness of social responsibility—trial may contribute to improved treatment for community in future?	Discuss notion of “collateral damage” between practitioners and between practitioners and patients/peers
Randomisation process	• Explain purpose of randomisation • Counter concerns that a computer is deciding on the fate of the patient, rather than a doctor • Address concerns relating to fairness of randomisation	• Reassure that regimens are also recommended by doctors • Build confidence of increased likelihood of a shorter regimen (not longer/worse than SoC) • Explain fairness of randomisation process (addressing concerns around bribes)	Communicate and explain how the randomisation process works in an accessible way
Consent process	Simplifying consent process	• Present information through conversation, using aide-memoire • Emphasise participant-led conversation	Prescriptive/formal questions are more likely to raise suspicion than understanding
Dual treatment approaches in one health system	Increasing MoH buy-in to expand access to eligible participants	Provide explanations for the different treatment approaches	• Explain the purpose and benefits of the CT • Offer opportunities to tour site if appropriate, to foster understanding and acceptability
Involvement of community doctors/MoH	Increase familiarisation, support, and trust in the CT	Collaboration between family/community doctors, policlinic	Create opportunities to engage with community doctors and the MoH to discuss the CT
Trust in CT, belief that this is about a better treatment	• Expand value of peer support for CT engagement • Build on achievement of the CT linked to positive values and quality of care	Enable potential CT patients to have access to CT wards rather than CC ward prior to initiating CT	Facilitate peer support to share experiential knowledge, quality of care and promote trust in new treatments
HCW confidence in CT	• Increase transparency around preliminary CT results • Increase familiarisation and exposure to CT among MoH HCW	Meetings with HCWs to share updates about the CT	Communicate regularly with all HCWs about the CT and share results when possible

The findings of this study provide insight into the experience of participation and recruitment into TB-PRACTECAL in Karakalpakstan, Uzbekistan. This has contributed to understanding and learning about patient investment in their clinical trial participation and what may help them value this more [24]. Although they are likely to be context-specific, generalised recommendations are made wherever possible and efforts will be made to ensure transferability to the other TB-PRACTECAL sites in Tashkent, Uzbekistan, Belarus and South Africa. Furthermore, the themes emerging from our analysis may inform clinical trials in low-resource settings within the wider TB community.

Strengths of this study include it being the first of its kind in Central Asia, documenting the experience of patient and practitioner perspectives in tuberculosis trials. We build upon preliminary qualitative findings to explore current arising themes in the trial. The methods used a patient-centred approach to recruitment, including process and tools, which optimises engagement with the trial. Additionally, a second researcher was involved in the data analysis to reduce the risk of researcher bias, and to minimise singular interpretation of the data.

Limitations are that it was not possible to recruit participants who had refused to join the CT. Individuals that were able to be contacted refused to participate or were deemed inappropriate as they were inpatients in the CC programme at the time of data collection.

The main recommendations from this research relate to enhancing trust in the trial through building familiarity and understanding, addressing fears and concerns, sharing preliminary results with HCW where possible, and supporting patients’ access to see the CT ward and to have contact with other CT patients. Additionally, it is important to address issues and concerns relating to CT uncertainty and the randomisation process, which appear to be key areas influencing CT refusal. There are successes too which need to be built upon and promoted such as compassionate and high quality of care.

This means that the recognition of systems as well as processual barriers need to be addressed through changing activities and approaches. We highlight (Table 4) key activities or approaches and practice changes that need to be addressed. Practical implementation requires devising these with the team and participant groups.

Finally, our qualitative analysis highlights ways in which insights developed together with patients and healthcare workers might inform approaches towards improved recruitment into trials, with the overall objective of delivering evidence for better treatments.

## Data Availability

The datasets generated and/or analysed during the current study are not publicly available but are available from on reasonable request; MSF has a managed access system for data sharing that respects MSF’s legal and ethical obligations to its patients to collect, manage and protect their data responsibly. Ethical risks include, but are not limited to, the nature of MSF operations and target populations being such that data collected are often highly sensitive. Data are available on request in accordance with MSF's data sharing policy (available at: http://fieldresearch.msf.org/msf/handle/10144/306501). Requests for access to data should be made to data.sharing@msf.org.

## Abbreviations

CC: Comprehensive care
CT: Clinical trial
FGD: Focus group discussion
HBC: Home-based care
HCW: Healthcare worker
IDI: In-depth interview
MDR-TB: Multidrug-resistant tuberculosis
MoH: Ministry of Health
MSF: Médecins Sans Frontières
OCA: Operational Centre Amsterdam
RCT: Randomised controlled trial
SoC: Standard of care
TB: Tuberculosis
TB1: Republican Centre of Phthisiology and Pulmonology (Hospital), Nukus
TB2: Republican Phthisiology Hospital No.2, Nukus
XDR-TB: Extensively drug-resistant tuberculosis
